# Stem cell competition in the gut: insights from multi-scale computational modelling

**DOI:** 10.1098/rsif.2016.0218

**Published:** 2016-08

**Authors:** Torsten Thalheim, Peter Buske, Jens Przybilla, Karen Rother, Markus Loeffler, Joerg Galle

**Affiliations:** 1 Interdisciplinary Center for Bioinformatics, Leipzig University, Haertelstr. 16-18, 04107 Leipzig, Germany; 2 Institute for Medical Informatics, Statistics and Epidemiology, Leipzig University, Haertelstr. 16-18, 04107 Leipzig, Germany

**Keywords:** mouse small intestine, clonal competition, Paneth cell specification, Wnt and Notch signalling, three-dimensional computational model

## Abstract

Three-dimensional (3D) computational tissue models can provide a comprehensive description of tissue dynamics at the molecular, cellular and tissue level. Moreover, they can support the development of hypotheses about cellular interactions and about synergies between major signalling pathways. We exemplify these capabilities by simulation of a 3D single-cell-based model of mouse small intestinal crypts. We analyse the impact of lineage specification, distribution and cellular lifespan on clonal competition and study effects of Notch- and Wnt activation on fixation of mutations within the tissue. Based on these results, we predict that experimentally observed synergistic effects between autonomous Notch- and Wnt signalling in triggering intestinal tumourigenesis originate in the suppression of Wnt-dependent secretory lineage specification by Notch, giving rise to an increased fixation probability of Wnt-activating mutations. Our study demonstrates that 3D computational tissue models can support a mechanistic understanding of long-term tissue dynamics under homeostasis and during transformation.

## 1. Introduction

The epithelium of the small intestine is one of the most rapidly regenerating tissues with a turnover time of only a few days. This permanent regeneration is enabled by intestinal stem cells (SCs) that reside at the bottom of small invaginations of the tissue, the intestinal crypts. In mice, about 5–15 of these cells have been identified per crypt under homeostatic conditions [1–3]. Elegant lineage tracing experiments demonstrated that they are actually capable of producing all types of differentiated cells found in the tissue [4]. The vast majority of these SC descendants are enterocytes (ECs) and two types of secretory cells, Paneth (PCs) and goblet cells (GCs). Their specification is largely controlled by Wnt- and Notch signalling (reviewed in [5]). These cells, while differentiating, move out of the crypt onto the villi, finger-like protrusions of the tissue, where they become shed. As an exception, PCs move down the crypt where they intermingle with the SCs and together with them form a SC niche [6]. The clones of all SCs compete for occupying this niche.

Such competition between SC clones represents a mechanism that potentially can eradicate mutant cells and their progeny from a tissue [7,8]. In the intestine, this competition leads to ongoing monoclonal conversion in all individual crypts of the tissue. As a result of this process, mutated cells become rapidly eliminated in most cases, i.e. the mutations do not become fixed. General features of this process have been studied experimentally [2,9,10]. However, the impact of the spatial organization of the intestinal crypts on the fixation of mutations remains largely unknown. We here demonstrate that three-dimensional (3D) computational crypt models can improve our understanding of these interdependencies.

The first individual cell-based computational model that enabled analysing spatial clonal competition in intestinal crypts *in silico* was introduced by Meineke *et al.* [11]. This type of model was subsequently extended by other groups [12]. Simulation results applying this type of model predicted that SCs located at the bottom of large intestinal crypts—even when subject to the same regulatory capabilities as those located above them—gain a significant competitive advantage, i.e. their clones have a higher chance to take over the crypt than others [13]. These results called into question the common assumption [14] that intestinal SC behaviour can be fully explained considering neutral competition between symmetrically dividing SCs [2,9]. Actually, intestinal SC clones do not take over the crypt with the same probability although the average probability of that event for a random selected clone is 1/*n*, where *n* is the number of SCs [15]. Recently, part of these model predictions on intestinal SC organization has been nicely validated by Ritsma *et al.* [10]. By quantitative analysis of clonal lineages in the small intestine, the authors demonstrated that SCs at the crypt base actually experience a competitive advantage over SCs at the border of the SC compartment.

Using a 3D individual cell-based model approach, we have recently simulated small intestinal crypt self-organization and explained: (i) robust tissue function under homeostasis as well as (ii) the consequences of loss and gain of function mutations regarding Wnt- and Notch signalling [16]. In these simulations, we noticed monoclonal conversion during homeostasis on timescales that agree very well with experimental observations. Here, we study how deregulation of Wnt- and Notch signalling affects this process.

It is known that more than 90% of human colorectal cancers (CRCs) show mutations in the Wnt pathway [17]. Mice with mutated adenomatous polyposis coli (APC), a negative regulator of Wnt signalling and frequent mutation target, rapidly develop adenoma in the small intestine and colon [18]. The cells of origin of these tumours are functional SCs of the crypts. Notch is activated in about 80% of human CRCs [19] and in many tumours from APC mutant mice [20]. In these mice, a synergy between Notch- and Wnt activation in tumour initiation has been demonstrated [20]. The cause of this synergy remained speculative. Recent experiments for the first time quantified the competitive potential of APC- and other mutant SCs experimentally [3,21]. We here ask how the organization of the SC niche affects this potential.

In the last decade, PCs, besides carrying out mucus secretion function [22], have been demonstrated to contribute to intestinal SC self-renewal and differentiation [6]. Presenting Notch ligands at their surface, these long-living cells ensure that neighbouring, undifferentiated cells become Notch-activated by receptor–ligand interaction and can remain in an undifferentiated state [23]. In addition, PCs secrete several soluble factors among them Wnt3a [6]. This Wnt3a secretion has been demonstrated to be sufficient to ensure Wnt activation in neighbouring SCs on a level required for their self-renewal. At the same time, PC specification itself is Wnt3a dependent [24]. According to these essential contributions to the organization of the SC niche, one can expect a strong impact of PC specification and distribution on clonal competition in the small intestine. However, this impact has not been investigated so far. Here, we use an extension of our 3D individual cell-based model to simulate spatio-temporal dynamics of clonal competition in mouse small intestinal crypts. Thereby, we focus on the impact of PCs on this process. We calculate fixation probabilities of mutations targeting Wnt signalling in intestinal SCs under wild-type and de-regulated Notch signalling. Based on our simulation results, we provide an explanation for the synergy of Wnt- and Notch signalling during tumour initiation and demonstrate that the fixation probability of mutations depends also on the process of how the mutations are acquired.

## 2. Material and methods

### 2.1. The three-dimensional crypt model

**Figure 1.**
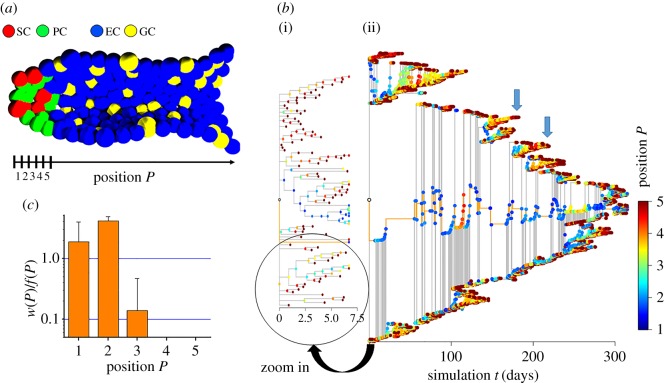
Crypt position controls clonal competition of SCs. (*a*) Sketch of the simulated system. SCs (red) are located at the bottom of the crypt being surrounded by PCs (green). Leaving the SC niche, SCs become committed either to the enterocytic (blue) or to the goblet lineage (yellow). The crypt position *P* is defined as the distance from the crypt bottom in cell radii (5 µm). (*b*) Typical phylogenetic trees of a wild-type crypt covering about one week (i) and 10 months (ii). Vertices are coloured according to the temporal position of the dividing SCs within the crypt. The root vertex (white circle) indicates the simulation start. The edges of the persistent clone are coloured in orange. An SC that is pushed to positions *P* > 2 often proceeds this movement until it reaches the border of the SC niche at position *P* = 5 and undergoes differentiation (arrows). (*c*) SCs at the very bottom of the crypt exhibit a competitive advantage, i.e. the ratio *w*(*P*)/*f*(*P*) (see text) is larger than one. Such a ratio was found for SCs at positions *P* < 3. Errors: s.d.

The basic structure of our computational model of the small intestinal crypt has been described in Buske *et al.* [16]. In short, the model incorporates explicit 3D representations of both the cells of the epithelium and the basal membrane. Cells are capable of forming contacts to neighbour cells and the basal membrane. They can move, grow and divide. Fate decisions are regulated by extrinsic stimulation of Wnt- and Notch signalling. The activities of these pathways control (i) whether a cell is capable of proliferating and (ii) into which lineage a cell becomes specified and eventually differentiated. Undifferentiated cells, i.e. SCs, are characterized by active Wnt- and Notch signalling (Wnt: high, Notch: high). The model considers specification of these cells into ECs (Wnt: low, Notch: high) and two types of secretory cells: PCs (Wnt: high, Notch: low) and GCs (Wnt: low, Notch: low) (figure 1*a*). The Wnt activity of a cell is assumed to depend on its position *P* along the crypt–villus axis, decreasing with the distance from the bottom of the crypt. Thus, an SC that moves up the crypt axis decreases Wnt signalling and specifies into an EC at a threshold position *P*_1_. The Notch activity of a cell is assumed to be determined by their neighbours. Secretory cells (PCs, GCs) are assumed to activate Notch signalling in neighbouring cells via ligand–receptor interactions, while they themselves are not capable of activating the pathway. Thus, an SC that loses PC contacts specifies into a PC due to reduced Notch signalling. In a similar process, ECs that lose GC contacts specify into GCs. The model assumes thresholds (*C*_1_ and *C*_2_) for the number of PC and GC contacts required for a stable SC and EC fate, respectively. All fate decisions are assumed to be reversible as long as the cells are not terminally differentiated [25,26]. Terminal differentiation is achieved if Wnt signalling falls below a second threshold value at position *P*_2_ > *P*_1_. As an exception, PCs become terminally differentiated at high Wnt activity, after having finished a last cell cycle subsequent to specification. As long as specified cells are not terminally differentiated, they are considered as progenitors and thus can proliferate. During proliferation, cells increase their volume with a defined growth rate until they reach twice their initial volume *V*_0_ and divide into two cells. Afterwards, the daughters start increasing their volume again if they are not subject to contact inhibition, i.e. are not compressed below a threshold volume *V_p_*. Cell movement is determined by adhesion, deformation and compression forces exerted by neighbouring cells or the basal membrane. In addition, ECs and GCs actively move up the crypt–villus axis and PCs down this axis. Cells that lose substrate contact undergo anoikis and are removed from the system. Cells that leave the crypt (*P* > 30) are removed as well. The reference parameter set applied in our simulations is given in the electronic supplementary material, table SA1.

### 2.2. Simulation of mutation events

In extension to the model described by Buske *et al.* [16], we enabled SCs (the cells-of-origin of intestinal tumours [18]) to accumulate mutations. We assumed that these mutations potentially affect the SC phenotype, i.e. change the cell growth time of the SCs or their sensitivity to activate Notch signalling, etc. In all cases, a mutation event was implemented as follows: after a steady-state cell turnover has been achieved, selected SCs are mutated once at a defined time point by changing the parameter set of them. The mutations are assumed to affect all selected SCs in exactly the same manner. Acquired mutations are passed on to each daughter cell. Analysis of a mutated system was started at the time point of mutation. Further details are given in electronic supplementary material, appendix S1.

### 2.3. Visualization and analysis of phylogenetic trees

**Figure 2.**
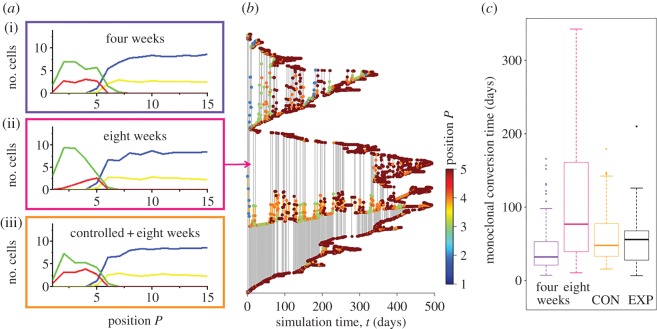
PC distribution affects monoclonal conversion. (*a*) Simulated long-term cell distributions for an intrinsically defined average PC lifespan of four (i) and eight weeks (ii). A long lifespan enables the PCs to accumulate at the bottom of the crypt leading to a depletion of SCs at this region. Additional extrinsic, cell contact-dependent control of PC lifespan can ensure that the cells intermingle also for intrinsically defined PC lifespan of eight weeks (iii). (*b*) Typical phylogenetic tree for an intrinsically defined PC lifespan of eight weeks. (*c*) Box plot of simulated monoclonal conversion times for an intrinsically defined PC lifespan of four and eight weeks and contact-dependent control of PC lifespan (CON). The contact-dependent control leads to monoclonal conversion times that agree with experimental observations. For a direct comparison, pseudo conversion times (EXP) have been generated that fit experimental results by Snippert *et al.* [2]. More details are given in electronic supplementary material, appendix S4.

Based on our simulation results, phylogenetic trees (cf. figures 1 and 2) have been generated with the package phytools [27] from the statistical analysis software tool R [28]. Phytools provides routines to generate phylogenetic trees and was extended by us with colourization of edges and vertices. Therewith, cell properties that change during the simulation can be visualized in the trees. Phylogenetic trees have been analysed to calculate the positional distribution of (i) SCs *f*(*P*), (ii) SCs whose clones won clonal competition *w*(*P*), and (iii) SC division events *p*(*P*). The results have been averaged over different simulations, and statistics have been derived.

## 3. Results

### 3.1. Stem cell position is a major competitive factor in the intestinal stem cell niche

As a first step, we aimed to test whether our computational model was capable of reproducing the experimental results recently provided by Ritsma *et al.* [10] on the competitive advantage of SCs at the crypt base. In simulation, we used the same parameter set as applied in our previous study ([16], compare electronic supplementary material, table SA1).

Figure 1*a* shows a cross-section through the simulated system. We used phylogenetic trees to visualize the simulated SC history. Examples are shown in figure 1*b*. In these trees, the edges represent SCs and each vertex represents a mitotic event of an SC. The length of an edge is defined by the individual cell cycle time. After cell division, the outgoing edges indicate the SC daughters arising from the mother. When the edges are orientated vertically, both daughters remain SCs during the next time step; otherwise, only one SC maintained the state. In the case of a leaf, both daughters became specified or underwent anoikis. In figure 1*b*, the vertices are coloured according to the position of the dividing SCs within the crypt. It can be seen that SCs located below position *P* = 3 (blue vertices) robustly stay in the crypt while those located above (yellow, red vertices) in most cases drift within a few days to the border of the SC compartment and become specified.

Owing to the competition between the SC clones, monoclonal conversion takes place over time, i.e. a single SC clone takes over the entire crypt. Subsequently, the clones originating from the cells of that clone for their part compete against each other and monoclonal conversion takes place again and so on. Retracing winning clones in our phylogenetic trees, we found that SC position within the crypt provides a competitive advantage. Figure 1*c* quantifies this advantage giving the ratio between the probability *w*(*P*) that a winning clone originates from position *P* and the fraction of SCs at position *P* at the starting point of competition, *f*(*P*). This ratio is either larger (advantage) or smaller (disadvantage) than 1. Consistent with the experimental results by Ritsma *et al.* [10], SCs at the very bottom of the crypt (*P* < 3) have a ratio *w*(*P*)/*f*(*P*) > 1 and thus a competitive advantage. Strikingly, in our crypts, position *P* = 1 does not have the highest ratio due to local cell compression leading to increased contact inhibition of growth and enhanced SC anoikis. The effect originates in the PC behaviour. If no PCs are present, SCs located at *P* = 1 are most competitive (see below) in agreement with simulation results obtained for the large intestine [13]. Our simulations suggest that our clonal analysis could be focused on the clones of the SCs located at positions *P* < 4 only, where *w*(*P*) > 0. Only the clones of these about 7 ± 1 SCs have the potential to take over the crypt under homeostasis due to the cell configuration at the starting point of competition. We call these SCs in the following ‘advantaged’ SCs.

We tested the relevance of the position-dependent competitive advantage by simulation of clonal competition between wild-type clones and clones with different mutations. In simulation series, we assumed that the SC located deepest in the crypt becomes mutated at a certain time point. Assuming that this cell starts dividing slower than all other SCs within the crypt, we defined a competitive disadvantage of the mutated cell that counteracts its position-dependent competitive advantage. We observed that its positional advantage can balance an increase of the cell cycle time by approximately 3–4 h, which is a biological relevant change as observed, e.g. in response to growth factors [29]. This balance was also obtained for changes in other parameters that are associated with a competitive disadvantage, e.g. a decrease in the capability to activate Wnt signalling [21]. Details can be found in electronic supplementary material, appendix S2. Thus, clonal position is a major competitive property in our model crypts. These results are robust for different crypt shapes. The competitive advantage of SCs at the bottom of the crypts is conserved in broader crypts. Details can be found in electronic supplementary material, appendix S3.

### 3.2. Paneth cell distribution affects the dynamics of monoclonal conversion

PCs move down the crypt while differentiating until they occupy their final position at the bottom of the crypt. Changes of their distribution are unavoidably accompanied by changes of the SC distribution. Thus, considering the above results, one can expect strong effects of the PC distribution on clonal competition. One potential modulator of the PC distribution is the PC lifespan.

In simulation series of our model, we actually found a strong dependence of PC distribution on their lifespan (*τ*^P^). An intrinsically defined average PC lifespan of four weeks resulted in stable intermingled states between PCs and SCs on long timescales (greater than 1 year). The PC turnover under these conditions required about eight weeks in agreement with experimental data [30]. In contrast, a PC lifespan of eight weeks resulted in nearly complete separation of PCs and SCs within a few months. Thereby, the PCs gradually overtook the bottom of the crypt. Only a few SCs remained at the border of the SC niche. PC turnover in this setting required more than 16 weeks. In figure 2*a*, the cell distributions for both scenarios are depicted. PC lifespans between four and eight weeks resulted in PC distributions that changed over time from that observed for four weeks to one similar to that observed at eight weeks. The time required for this drift decreased from more than 1 year (*τ*^P^ = four weeks) to a few months (*τ*^P^ = eight weeks).

Analysing the phylogenetic trees of these simulations, we found that the differences in the PC lifespan and the related changes in the cell distributions strongly affected clonal competition. Robust monoclonal conversion within, on average, six weeks was observed for a PC lifespan of about four weeks. Assuming a PC lifespan of eight weeks, clonal competition between the remaining SCs at the border of the SC niche was nearly abolished and two or more clones often survived for more than six months. Figure 2*b* shows a typical phylogenetic tree for an intrinsically defined PC lifespan of eight weeks. Here, two clones coexist for about 1 year. More details on the clonal competition can be found in the electronic supplementary material, appendix S4. Our results suggest a regulated lifespan of PCs of about four weeks. Thereby, the trigger of cell death remains speculative. Specific modes of programmed cell death of PCs have been identified. However, the inducers of them have still to be determined [31].

We hypothesized that the PC lifespan is at least in part environmentally controlled. In simulation series, we assumed that PCs undergo programmed cell death if they do not form contacts with SCs for more than 12 h. To avoid artificially long-living PCs, we combined the mechanism with an intrinsically defined PC lifespan of eight weeks. As shown in figure 2*a*, this assumption (keeping all other model parameter unaffected) led to self-organization of the system such that all cells distribute similar to the case of PCs with an intrinsically defined lifespan of four weeks. Thereby, more than two-thirds of the PC death events were triggered by the contact mechanism. The PC distribution was stable for more than 1 year. In this setting, the average time for monoclonal conversion robustly adjusted to about eight weeks with a variation close to experimental observations (figure 2*c*, see fig. 6*c* in [2]). We used this setting in all following simulations.

### 3.3. Autonomous Notch signalling provides a competitive advantage and accelerates monoclonal conversion

Cell intrinsic activation of Notch signalling, here referred as ‘autonomous Notch’, suppresses specification of secretory cells and thus leads to PC and GC depletion [32]. Such activation can be achieved by forced expression of the Notch intracellular domain (NICD) [33]. We next studied the consequences of autonomous Notch on the monoclonal conversion.

**Figure 3.**
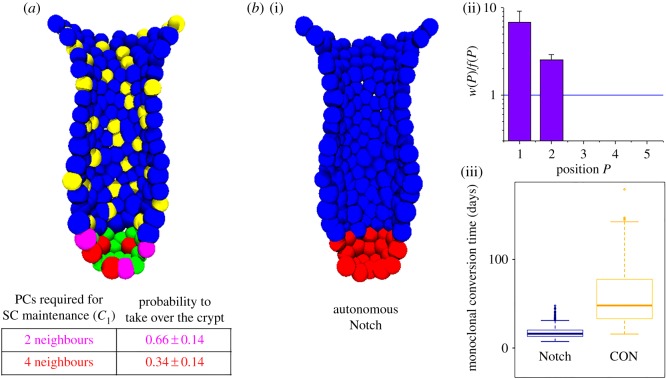
Autonomous Notch provides a competitive advantage. (*a*) Sketch of the simulated competitive scenario. SC maintenance requires either *C*_1_ = 2 (magenta SCs) or 4 (red SCs) Notch-ligand expressing neighbours. The SCs, which are less dependent on external Notch activation, have a competitive advantage. They take over the crypt in about 66% of the simulations. (*b*)(i) Sketch of the simulated system for *C*_1_ = 0 (and *C*_2_ = 0). PCs (and GCs) are no longer specified. In this case, the most competitive SCs originate from position *P* = 1 (ii, errors: s.d.). The time to monoclonal conversion (iii) decreases from about six weeks under wild-type conditions (CON) to less than three weeks. More details are given in electronic supplementary material, appendix S4.

In our former study [16], we assumed that wild-type SCs (ECs) require a minimal number *C*_1_ = 3 (*C*_2_ = 1) of Notch-ligand expressing neighbours, i.e. PCs (GCs), to suppress specification into a secretory fate [23]. In the present study, we modified these conditions implementing a competitive scenario. We assumed *C*_1_ = 4 for one half of the SCs and *C*_1_ = 2 for the other. A cross-section through a start configuration of these simulations is shown in figure 3*a*. In the simulations, we considered SC self-renewal to be independent of PC-secreted Wnt3a, i.e. we assumed that Wnt3a secretion by external sources in the SC niche was sufficient to guaranty SC maintenance [24]. Moreover, we assumed a fixed size of the intestinal SC niche, independent of *C*_1_. We observed a clear competitive advantage for SCs being less dependent on PC neighbours. This can be explained by the fact that stronger dependence on PCs requires more frequent specification into this lineage at the expense of SC maintenance.

Obviously, decreasing *C*_1_ leads to a decrease in the number of PCs in the SC niche. Because of the fixed size of the niche, this is accompanied by an increase in the number of SCs and thus by a decreased average probability of a randomly selected SC clone to take over the crypt (1/*n*). In our simulations, we found that in parallel the time for monoclonal conversion decreased as well. For *C*_1_ = 0, the entire SC niche becomes occupied by SCs only (figure 3*b*). We found that in this case the time required for monoclonal conversion decreased to less than three weeks. Thereby, position *P* = 1 provided the highest competitive advantage as predicted by Mirams *et al.* [13]. The number of ‘advantaged’ SCs in these crypts was about 11 ± 1. Figure 3*b* shows simulation results for *C*_1_ = *C*_2_ = 0, where autonomous Notch in the SCs is passed on their progeny.

### 3.4. Autonomous Notch signalling increases competitive potential of Wnt mutants

As mentioned above, when one randomly selects and labels an SC, the average probability of its progeny to take over the crypt will be 1/*n*, where *n* is the number of SCs. In principle, any neutral mutation, which does not provide a competitive (dis-)advantage, can be considered as a label. The average probability that such a mutation becomes fixed in the system is 1/*n* whereas its elimination probability is 1 − 1/*n*. Thus, in model crypts with 13 SCs about 92% of the neutral mutations in SCs are lost due to ongoing clonal competition. Reducing the analysis to the seven ‘advantaged’ SCs that can take over the crypt, still 86% of the SC mutations become lost.

In our model, autonomous Notch increases the number of SCs and thus decreases the fixation probability of subsequently acquired mutations. However, due to the increased SC number, the number of acquired mutations per time unit increases as well. Thus, the average number of mutations becoming fixed per time unit and the crypt remains constant with respect to wild-type crypts. However, this is no longer valid if Notch activation affects the competitive properties of the mutated cells.

**Figure 4.**
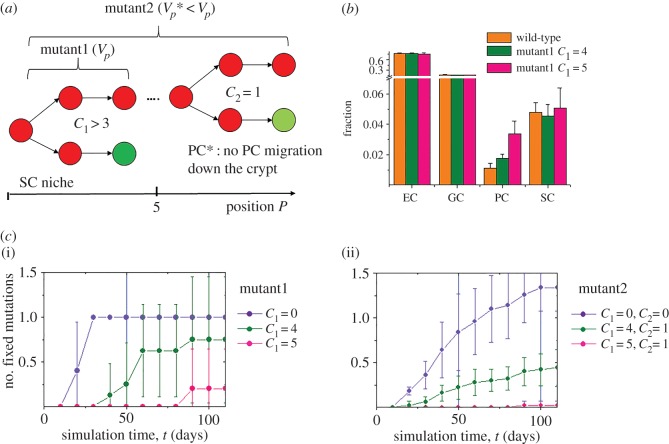
Fixation of Wnt-activating mutations depends on Notch. (*a*) For mutant1 SCs, the number of Notch-ligand-expressing neighbours required for SC maintenance *C*_1_ was increased to values larger than 3. Mutant2 SCs in addition were assumed to be maintained outside the SC niche, to have a lower sensitivity to contact inhibition of growth (V_p_* = 0.76 *V_p_*, electronic supplementary material, table SA1) and to specify into PCs that lack a migration bias. (*b*) The fraction of cells produced by wild-type and mutant1 SCs. Shown are average cell-type fractions within the progeny of 10 wild-type and mutant SCs. (*c*) (i) The increased PC requirement (pink symbols: *C*_1_ = 5, olive symbols: *C*_1_ = 4) by mutant1 SCs led to a competitive disadvantage of them, which vanishes in the case of autonomous Notch (blue symbols: *C*_1_ = 0). (ii) Mutant2 SCs (here: *C*_2_ = 1) show a competitive advantage that becomes even larger in the case of autonomous Notch (*C*_1_ = 0, *C*_2_ = 0). All errors: s.d.

It has been shown that Wnt expression induces PC differentiation cell autonomously [24]. This implies that mutants with activated Wnt signalling, as APC loss-of-function mutants, preferentially specify into the Paneth property. Accordingly, the competitive potential of these mutants is reduced, i.e. PC specification acts as a suppressor of Wnt-activating mutations. To quantify the effects of Notch signalling on the fixation of Wnt-activating mutations, we simulated the Wnt-dependent PC specification described above assuming that mutant SCs require *C*_1_ > 3 PCs for SC maintenance (mutant1, figure 4*a*).

As expected, due to the increase of *C*_1_, mutant1 SCs (*C*_1_ = 4, = 5) produced more PCs compared with wild-type SCs (*C*_1_ = 3, figure 4*b*). Accordingly, on a wild-type background, they were found to be less competitive, i.e. out of *n* mutant1 clones less than 1 became fixed. Videos of selected simulations are given in the electronic supplementary material, video S1a,b. This competitive disadvantage vanished on an autonomous Notch background, in particular if *C*_1_ = 0 applies to both mutant and wild-type SCs (figure 4*c*).

However, experiments showed that mutations activating Wnt signalling, as APC loss-of-function mutations, are associated with a competitive advantage [21]. So, in additional simulation series, we assumed that the mutated SCs in addition keep their proliferation activity throughout the crypt and specify in PCs that lack the migration bias towards the bottom of crypt [34]. Moreover, they are less sensitive to contact inhibition of growth to enable hyper-proliferation [18] (mutant2, figure 4*a*). Mutant2 clones on a wild-type background actually showed a competitive advantage as observed for APC mutants *in vivo* [21]; i.e. more than one out of *n* mutant2 clones became fixed over time (figure 4*c*). Importantly, mutant2 SCs were also ‘advantaged’ at positions *P* > 3. Moreover, these mutants, while expanding throughout the crypt, strongly blocked migration outwards the crypt. As a consequence, wild-type SCs frequently remained in crypts that were dominated by a mutant clone and became contact inhibited. Thus, monoclonal conversion did not occur. A representative video is given in the electronic supplementary material, video S2. Nevertheless, also for mutant2 SCs, the number of fixed mutants on an autonomous Notch background (*C*_1_ = 0) was higher than that found on a wild-type background (*C*_1_ = 3). After 50 days about three-times more mutant2 clones became fixed.

Thus, our model predicts that in the case of autonomous Notch, Wnt-activating mutations are fixed more frequently increasing the risk of further tissue transformation. This might explain the experimental findings of Fre *et al.* [20], demonstrating a synergy between the Notch- and Wnt pathway in triggering tumourigenesis in mice.

### 3.5. Autonomous Notch signalling supports accumulation of cell division-associated mutations

**Figure 5.**
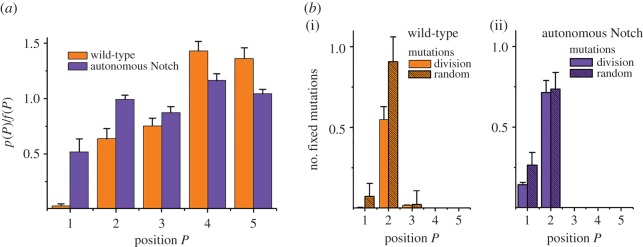
Fixation of cell division-associated mutations depends on Notch*.* (*a*) Distribution of cell division events. Shown is the ratio between the probability *p*(*P*) to find a cell division event at position *P* and the fraction of SCs located at this position *f*(*P*). Cell division events are less frequent at positions *P* < 4 for wild-type and *P* < 2 in the case of autonomous Notch. (*b*) As a consequence, the distribution of division-associated mutations changes and thus also their fixation probability. For wild-type crypts (i), the sum over all division-associated mutations that became fixed out of *n* independent mutations (plain orange) reduces to about half the value (0.57) observed for random distributed mutations (striped orange, 1.00). Under autonomous Notch (ii), this sum increases to 0.86 (plain blue). Errors: s.d.

For the scenarios described above, we assumed that the mutation process is random and in particular position independent. Thus, the distribution of mutations equals that of the SCs *f*(*P*). When mutations are coupled to the cell division process, highly proliferative cells become more frequently mutated than their quiescent neighbours. Setting the distribution of mutations equal to the distribution of SC division events *p*(*P*), it will deviate from the cell distribution *f*(*P*). Related simulation results for the ratio *p*(*P*)/*f*(*P*) are shown in figure 5*a*.

In wild-type crypts, SCs at *P* < 4 divide less frequently than SCs at the niche boundary, mainly due to contact inhibition of growth. This is in agreement with experimental findings [35]. As a result, mutations are most frequently observed at the boundary of the SC niche. Clones that originate at the boundary are lost frequently, and thus, the fixation probability of mutations in these systems is lower compared with randomly mutated cells. Figure 5*b* shows the numbers of mutations becoming fixed out of *n* independent mutations. For division-associated mutations, we observed in total 0.57 (figure 5*b*) fixed mutations, i.e. the fixation probability was decreased by a factor of about two compared with random distributed mutations (1 fixed mutation).

Considering autonomous Notch, PCs vanish and accordingly the distribution of cell division events *p*(*P*) and that of cells *f*(*P*) changes. Results for *p*(*P*)/*f*(*P*) are shown in figure 5*a*. In this case, the ratio *p*(*P*)/*f*(*P*) is close to 1 for positions *P* > 1. In particular, mutations at position *P* = 2 are as frequent as for random mutations. Accordingly, we found that in this system the effect of non-random distribution of mutations nearly vanishes, and the total number of fixed mutations is again close to 1 (figure 5*b*). Thus, our model suggests that autonomous Notch results in a higher probability of accumulation of cell division-associated mutations compared with wild-type Notch regulation.

## 4. Discussion

### 4.1. Effects of stem cell position

In agreement with previous simulation studies [13] and with recent experimental results [10], we observed a strong competitive advantage for SCs being located at the bottom of the crypt. The advantage is observed for identical regulated SCs and thus originates exclusively from the spatial organization of the crypt. Accordingly, an SC from higher positions can overtake this advantage just by changing the position within the crypt. Our simulations enable calculation of the number of ‘advantaged’ SCs, i.e. the number of SCs required so that the clones can take over the crypt starting with a defined SC niche configuration. In a wild-type crypt, this number is about seven and thus lower than the number of crypt base columnar cells (10–14) expressing the LGR5 stem cell marker [14]. All SCs located above position 3, in particular so-called+4 SCs, form a reservoir of potential SCs that can be activated on demand [36].

In our simulations, position *P* = 2 provides the highest competitive advantage under normal homeostatic conditions. SCs located at position *P* = 1 partly undergo contact inhibition of growth and anoikis and are thus less competitive. This behaviour originates in the dynamic properties of PCs that experience a constant migration bias downwards in the crypt in our model. The underlying EphB/ephrin-B signalling can be experimentally manipulated [37]. Computational approaches tackling this problem have been introduced [38]. Nevertheless, its effects on SCs competition are largely unknown.

### 4.2. Effects of Paneth cell distribution

SC competition also depends on the quasi-stationary-distribution of the PCs. We have shown that strong accumulation of PCs at the bottom of the crypt would nearly abolish competition between the remaining SCs. This behaviour occurs in our model for a long PC lifespan. Experimentally such local SC depletion has been observed following massive telomere dysfunction [39] but not during normal tissue homeostasis. We suggested that during homeostasis, PCs undergo programmed cell death if they do not form contacts with Notch-receptor-expressing cells. Under this assumption, the PC distribution and turnover self-organizes such that it fits experimental data [30,40]. One might speculate that Notch ligands that are not bound by Notch receptors present on SCs contribute to differentiation of innate lymphoid cells [41], which in turn express interferon gamma, known to induce PC programmed cell death [42].

Alternative mechanisms controlling PC distribution might be specific PC–SC adhesion mechanisms or de-differentiation of PCs, as observed during damage repair [25]; the latter being unlikely due to its rare occurrence during homeostasis. The question remains whether such mechanisms can also trigger PC death. Further experiments are required to unravel these processes.

In our simulations, we focused on clonal competition in the mouse small intestine. In addition, recently, models of SC organization in the large intestine have been introduced [13,43–45]. In this system, where no PCs are present, the time for monoclonal conversion is much shorter than in the small intestine [46]. We observed decreased conversion times in the small intestinal crypt following intrinsic Notch activation. This suggests that the PC equivalent cells observed in the large intestine [6] behave like SC. Consistently, data of the mouse large intestine show no decrease in the proliferation index at the bottom of the crypts, suggesting that these cells proliferate [47].

### 4.3. Modelling Wnt signalling and mutations

In our simulations, SC maintenance requires that the SC niche is sufficiently supplied with Wnt3a. According to findings by Farin *et al.* [24] that PC-related Wnt3a is dispensable for SC maintenance *in vivo,* this might be ensured *in vivo*. Interestingly, PC-independent intestinal SC homeostasis has been observed after complete ablation of PCs [48,49], suggesting an independence also from PC-induced Notch activation. However, this ablation has been achieved by knock-down of the transcription factor Atoh1, which is essential for specification of the secretory fate. Accordingly, these results have been suggested to document independence of intestinal homeostasis from PC-related Wnt3a production and Notch activation if specification into the secretory fate is suppressed [48].

In our model, this complex signalling is strictly simplified. Wnt signalling depends on the position of the cells along the crypt–villus axis only and is decoupled from Notch signalling. Accordingly, the fate of the cells is changed if they cross defined positions (*P*_1_, *P*_2_). Computational approaches have been suggested that consider transcriptional regulation of Wnt targets in much more detail [12]. These approaches also provide the opportunity to model different types of mutations of the pathway. However, the problem remains how to map the de-regulated expression states onto cellular phenotypes. In an alternative approach, crypt cell parameters, as e.g. the cell cycle length, have been described as emergent properties of gene regulatory networks [50]. Following this strategy, mutations of the network immediately result in phenotypic changes as well. We here modelled well-documented phenotypic consequences of Wnt mutations by changing cell parameters directly.

### 4.4. Effects of Notch signalling on the fixation of mutations

Autonomous Notch has been found to be supportive in tumourigenesis (reviewed in [51]). We have demonstrated that these effects might originate in substantial changes of the fixation probability of subsequently acquired mutations. Such a scenario is given when Notch activation changes the competitive properties of the subsequently mutated SCs. We predict that Notch in particular supports fixation of mutations activating the Wnt pathway. In fact, autonomous Notch suppresses Wnt-dependent PC specification. Accordingly, the Wnt mutations can become fixed more easily and early states of tumour development as metaplasia and adenoma can occur more frequently. This is in agreement with experiments showing that the activation of Notch by induced expression of NICD led to a strong decrease of the survival time of mice heterozygous for a loss of function allele of APC [20].

A second scenario demonstrating the oncogenic potential of activated Notch is given when mutation events are preferentially associated with cell division events. As Notch activation and related PC depletion changes the distribution of proliferation events, it will in this case also change the distribution of mutations. Thereby, Notch activation in particular increases the frequency of mutations in SCs at the bottom of the crypt and thus the frequency of their fixation. Such a scenario might be given for cells with mismatch repair deficiency [52]. Accordingly, we expect Notch activation to play a role in the initiation of hereditary non-polyposis CRC.

### 4.5. Three-dimensional *in silico* simulations

In the last years, different computational approaches have been introduced that enable simulation of tumour initiation and progression in the intestine (reviewed in [53,54]). Simulations of scenarios that address deviations from the 1/*n* fixation probability following Notch activation require an explicit 3D representation of the intestinal crypt. Moreover, they require simulation of lineage specification. Other computational models of tumour initiation and progression so far neglect these features [55,56].

Clearly, most scenarios discussed above potentially will change the geometry of the crypt. We have shown that altered crypt geometry affects the timescales of the competition processes, while the qualitative behaviour remains largely conserved. However, all these simulations assumed a fixed geometry. Simulations explicitly considering flexible tissue shape would require a more sophisticated model. Such a model has been introduced by us recently for intestinal organoids [57,58]. Applying this kind of model to intestinal crypts will allow simulation of crypt fission events and therefore of clonal competition during adenoma formation. Such simulations will further support our understanding of the impact of clonal competition on tumour initiation and progression, a field of high clinical relevance that is currently not well understood [59].

## 5. Summary

We have applied a 3D individual cell-based model to study clonal competition in intestinal crypts. Thereby, we focused on the 3D structure of the small intestinal SC niche being composed of undifferentiated functional SCs and PCs. In simulation series, we demonstrated that both cell distribution and niche composition have a strong impact on clonal competition and thus on monoclonal conversion of the crypts. As a consequence, the efficiency of tumour initiation by mutation fixation is strongly affected by such changes. Potential sources of them are pathological changes of signalling pathways controlling SC self-renewal and lineage specification in the small intestine. As an example, we analysed the impact of Notch and Wnt signalling, two major SC signalling components, on clonal competition in the crypt. Our results provide a mechanistic explanation of the experimentally observed synergy between activated Notch and Wnt signalling in initializing tumour formation.

In general, our study exemplifies the potential of 3D computational models in supporting a mechanistic understanding of long-term dynamics of regenerative tissues. Such models, when they have been tested to consistently describe a large number of experimental results on tissue homeostasis, enable substantial hypotheses on the systems behaviour under perturbations and during tissue transformation. Therewith, they can guide future experimental studies and help to optimize their design.

## Supplementary Material

Electronic Supplementary Material

## Supplementary Material

supp_figure_A1

## Supplementary Material

supp_figure_A2

## Supplementary Material

supp_figure_A3
