# Supplementary material for: Stem cell competition in the gut: insights from multi-scale computational modelling
Source: J R Soc Interface. 2016 Aug;13(121):20160218. doi: 10.1098/rsif.2016.0218 (PMC5014057; doi:10.1098/rsif.2016.0218)
Supplement: Electronic Supplementary Material [file rsif20160218supp1.pdf]

Table A1. Parameter set of the reference crypt model (Buske et al. 2011)

Symbol	Value	Parameter	References
**Parameter of the cell model**			
V_0	$4/3\pi\ (5\mu m)^3$	minimal volume of an isolated cell	estimated
τ	14 h	cell growth time	results in an effective cell cycle time ~24h
E	1kPa	Young modulus	Galle, Loeffler & Drasdo (2005)
ν	1/3	Poisson ratio	
ε_c	200 µN/m	cell-cell anchorage	
V_p	$0.88\ V_0$	threshold volume for contact inhibition	set
**Parameter of the BM model**			
z_0	150 µm	length of the crypt	set, according to measured properties of the crypt shape (Potten and Loeffler, 1990)
r_0	60 µm	crypt radius at the crypt-villus junction	
λ_1	0.25	shape parameter 1	
λ_2	0.1	shape parameter 2	
λ_{MAX}	1.25 µm	maximum in-radius of a network triangle	set (technical)
Ω	0.95	optimal cell-BM distance in cell radii	set
ε_K^{Paneth}	$35\ 10^{-12}$ Nm	maximum cell-knot interaction energy of PCs	set
ε_K^{other}	$5.5\ \ 10^{-12}$ Nm	maximum cell-knot interaction energy of all other cells	ensuring apoptosis rates <5% (Marshman et al., 2001)
**Parameter of crypt dynamics**			
η_c	$5\ 10^{10}$ Ns/m³	friction constant for cell-cell friction	Galle, Loeffler & Drasdo (2005)
η_{BM}	3.2 Ns/m	friction coefficient for cell-BM friction	fit: turnover
η_{VO}	400 Ns/m	friction coefficient regarding volume changes	Galle, Loeffler & Drasdo (2005)
F_A^{Paneth}	7.5 nN	active migration force of PCs	fit: distribution of PCs
F_A^{other}	4.5 nN	active migration force of all other cells	fit: turnover and Brdu data
**Parameter of the lineage specification and differentiation model**			
P_1	-125 µm	position of the Wnt- threshold TP_{Wnt} for priming	fit: size of the PC compartment
P_2	-87.5 µm	position of the Wnt- threshold TD_{Wnt} for differentiation	fit: turnover and Brdu data
C_1	1	minimum number of ligand expressing neighbors in the niche	set: refers to TD_{Notch} /LP^{Paneth} and TD_{Notch}/LP^{Goblet} in Buske et al. (2011)
C_2	3	minimum number of ligand expressing neighbors outside the niche	
τ^P	8 weeks	average PC lifespan with contact to SCs	see text
τ^A	12 h	max. PC lifespan without contact to SCs	see text

Appendix1: Simulation of Wnt- mutants

We performed five simulations for each mutation scenario. In each simulation, the crypt configuration during the mutation event is replicated N times so that each SC can be mutated individually whereas the other SCs remain unchanged in the respective replicate. Hence one initial wild type configuration will yield to N individual mutation configurations. Afterwards each mutation configuration is simulated independently, thus 5N simulations have been performed as each SC in each configuration has been selected for mutation.

Videos A1a,b and A2 show examples of this kind of simulations for mutant1 and mutant2 cells, respectively. In these videos mutated SCs and their progeny are colored as following: SC (pink), PC (cyan), GC (black) and enterocytes (white). Videos A1a,b have the same initial configuration, but differ in the SC selected for mutation. In Video A1a the selected SC clone overtakes the crypt in approximately 13 weeks, while in Video A1b the selected SC clone vanishes after about 2 weeks. Both videos use a bottom view. In Video A2 the selected SC clone spreads very fast and overtakes the entire crypt in less than 3 weeks. This video uses a side view. Note, that in each simulation terminal differentiated wild type PCs may persists even after conversion.

Additional references in the Appendix:

Galle J, Loeffler M, Drasdo D. (2005). Modeling the effect of deregulated proliferation and apoptosis on the growth dynamics of epithelial cell populations in vitro. Biophys J., 88:62-75.

Marshman E, Ottewell PD, Potten CS & Watson AJ (2001). Caspase activation during spontaneous and radiation-induced apoptosis in the murine intestine. J. Pathol. 195, 285-92.

Potten CS & Loeffler M (1990). Stem cells: attributes, cycles, spirals, pitfalls and uncertainties. Lessons for and from the crypt. Development 110, 1001-20.

Appendix2: SC position as a competitive factor

In order to demonstrate the relevance of SC position regarding SC competition we simulated competition between wild type SCs and a mutated SC that is located at the very bottom of the crypt. Thereby, we checked the extent to which the mutated cell can balance an intrinsic competitive disadvantage Fig. A1 shows results of simulations assuming that the mutated cell has a changed cell growth time τ or a changed threshold position P_1 of the Wnt-activity; the latter referring to a changed capability to activate Wnt-signaling at a given external Wnt-concentration.

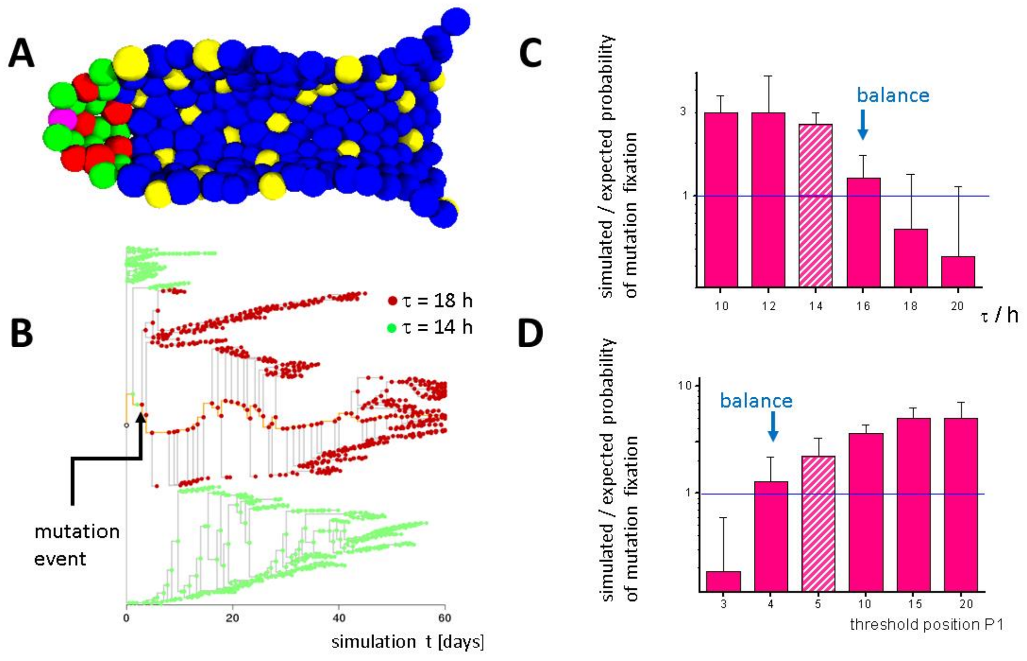

Fig A1. SC position balances mutation-associated competitive disadvantage. A) Sketch of the simulated system. In all simulations the SC with the lowest position (magenta cell) becomes mutated. B) Phylogenetic tree of a selected simulation assuming that the mutated SC has a 4 hours longer cell growth time τ than the wild type SCs. Vertices are colored according to the cell growth time of the dividing cells. After about 8 weeks the mutated SC clone (dark red) overtakes the niche. C,D) Ratio between simulated and expected probability of a mutation to become fixed assuming a mutated cell growth time τ (C) and Wnt-threshold position P_1 (D). The mutated SC at the bottom of the crypt has a competitive advantage until its growth time τ is set to about 1.2 times the value of wild type SCs, resulting a 3-4 hours longer cell cycle time. Alternatively, it can balance a change of threshold position P_1 to 4. Reference parameter simulations are indicated by white stripes.

Appendix 3: Clonal competition in crypts with different shapes

In order to analyze the impact of the explicit crypt shape on clonal competition we generated a set of crypts with different length, width and curvature (Buske et al. 2011) and compared the results with those obtained for the crypt used throughout the study (here called: reference crypt). Fig. A2 shows an example of a short and broad crypt that contains approximately the same number of cells as the reference crypt. Model parameters of the broad crypt are given in Table A2.

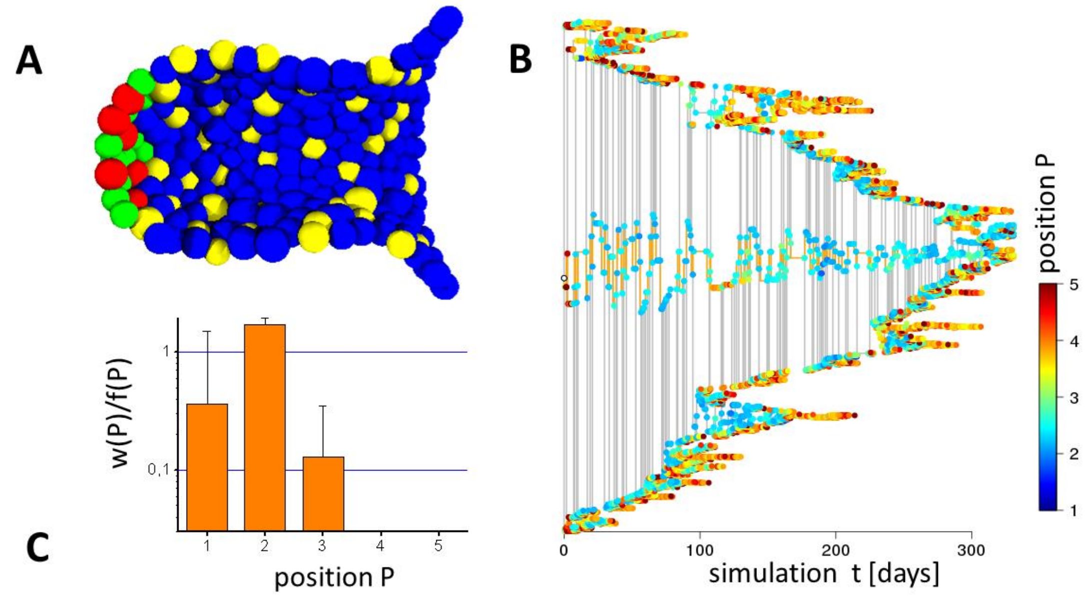

Fig. A2: Clonal competition in broad crypts. A) Sketch of the simulated system. Keeping similar cell numbers as the reference crypt, the 'broad' crypt has about 4/3 times the diameter of the reference crypt. B) Phylogenetic tree of a selected simulation. Vertices are colored according to their position within the crypt. Times of clonal conversion are similar to the reference crypt. C) SC at the bottom of the crypt (P=2) have a competitive advantage, i.e. the ratio w(P)/f(P) (see text) is larger than one.

Table A2. Model parameters of the broad crypt

Symbol	Value	Parameter
**Parameter of the BM model**		
z_0	130 µm	length of the crypt
r_0	80 µm	crypt radius at the crypt-villus junction
**Parameter of the lineage specification and differentiation model**		
P_1	-115 µm	position of the Wnt- threshold TP_{Wnt} for priming
P_2	-67.5 µm	position of the Wnt- threshold TD_{Wnt} for differentiation

Appendix 4: Monoclonal conversion dynamics

During the simulation all SCs present at a selected time point have been labeled. The number of the individual clones present at later times and their size have been calculated using the information of the phylogenetic trees. As all progenitors are capable to de-differentiate into SCs (van Es et al., 2012; Tetteh et al., 2016) we analyzed clones containing SCs and their direct progeny.

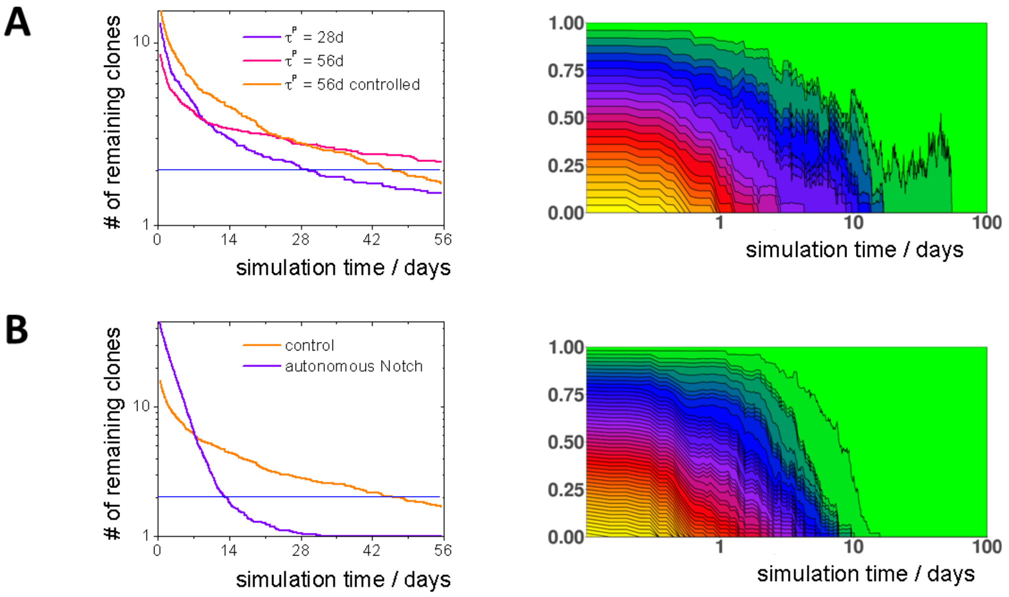

Fig. A3: Individual clone properties during competition. A) The PC lifespan and distribution strongly affects the monoclonal conversion time. (Left panel): Number of initially labeled SC clones remaining in the crypt over time. At t=0 all SCs have been labeled. Shown is an average over 50 realizations of the system for a PC lifespan of 4 (blue), 8 weeks (red) and for contact controlled PC lifespan (orange). (Right panel): Example of SC competition in case of a contact controlled PC lifespan. Shown are the clones of all SCs that were present in the crypt at time t (each with a different color). The clones comprise SCs and their direct progeny. Monoclonal conversion takes place after about 55 days (green clone). B) Autonomous Notch accelerates monoclonal conversion. (Left panel): As is A), shown is an average over 50 realizations of the system for autonomous Notch (C_1=0, dark blue) and for the reference system (C_1=4, orange). (Right panel): Example of SC competition under autonomous Notch. Shown are the clones of all SCs that were present in the crypt at time t (each with a different color). They comprise SCs and their direct progeny. Monoclonal conversion takes place after about 16 days (green clone).
