# Supplementary figures and images for: Stem cell competition in the gut: insights from multi-scale computational modelling
Source: J R Soc Interface. 2016 Aug;13(121):20160218. doi: 10.1098/rsif.2016.0218 (PMC5014057; doi:10.1098/rsif.2016.0218)

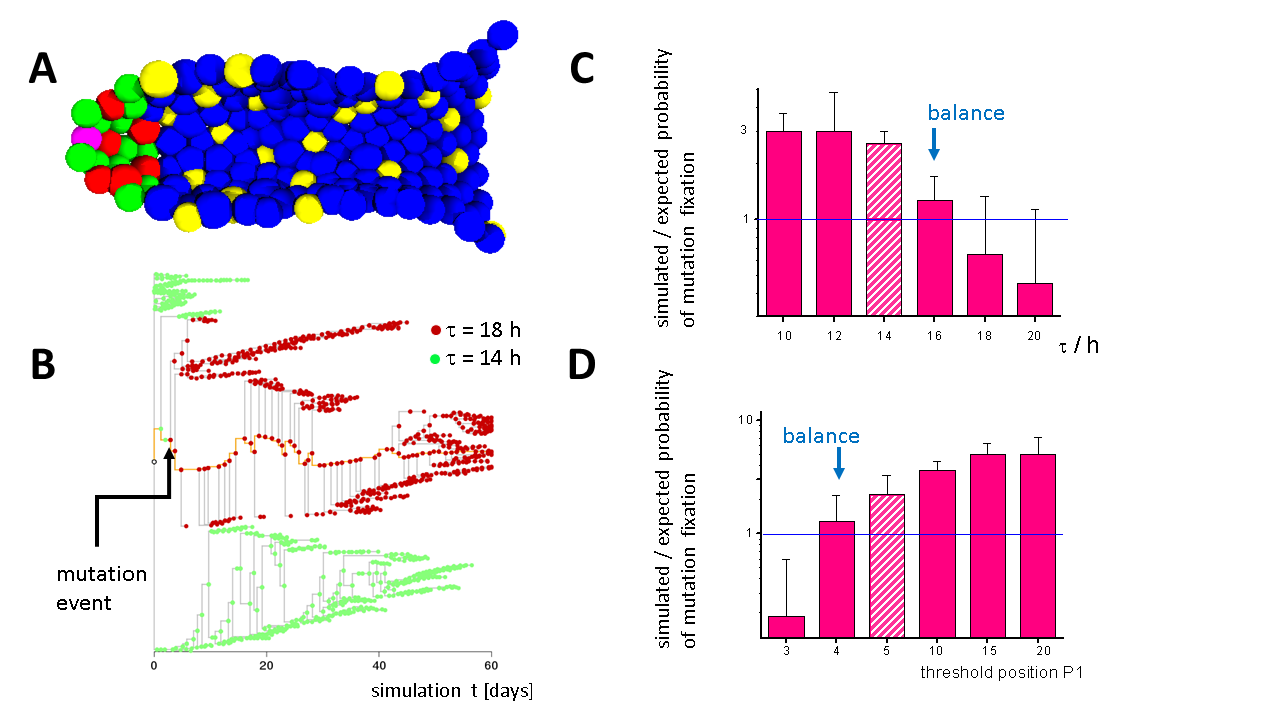

Supplement: supp_figure_A1 [file rsif20160218supp2.tif]

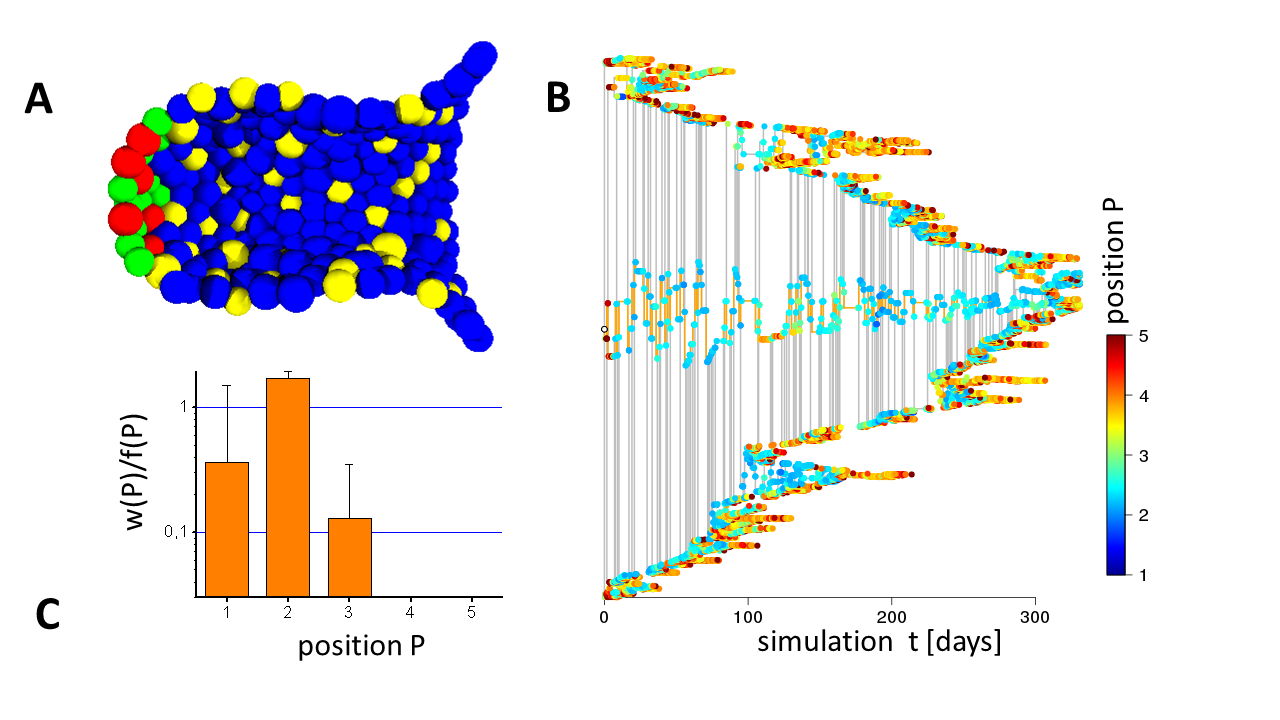

Supplement: supp_figure_A2 [file rsif20160218supp3.tif]

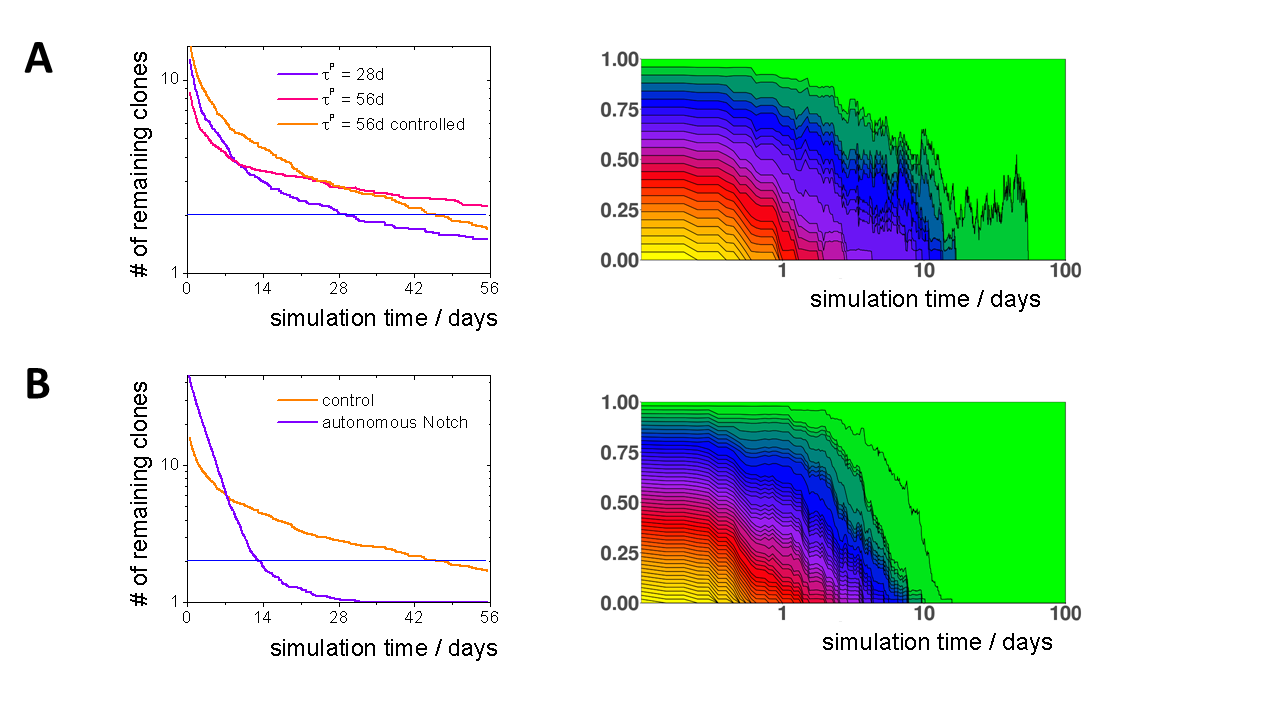

Supplement: supp_figure_A3 [file rsif20160218supp4.tif]
